# Dr. Wainwright’s Book

**Published:** 1908-12

**Authors:** 


### EDITORIAL DEPARTMENT.

### DR. WAINWRIGHT'S BOOK.

### THE MEDICAL AND SURGICAL KNOWLEDGE OF WILLIAM SHAKSPERE.

We have been favored with a presentation copy of Dr. Wainwright's delightful little book, entitled *"The Medical and Surgical Knowledge of William Shakspere."*

It is a gem. Dr. Wainwright has rendered a most acceptable service to the medical profession and to all students of literature by culling out and putting in pleasing form nearly or quite all of the passages in Shakespeare which show that *the writer* of those wonderful dramas had a broad and deep knowledge of medicine; just as Lord Campbell did that the *writer* had a deep and comprehensive knowledge of law, and just as others have done that the writer had a deep and extensive knowledge of science, literature and art, of history, travel and arms, and even of Greek and Latin, of all of which "Shakspere," as Dr. Wainwright prefers to call him, was profoundly ignorant.

The credulity of those who believe that an almost totally illiterate man, as William Shakespeare evidently was, could have produced the immortal works that bear his name; which astonished the world, and are still the admiration and wonder of the age, is of a robust kind,—a credulity that is little else than blind and unreasoning faith in miracles. I am not of those who believe or are willing to admit that a man who "never owned a book, or ever saw one" (Theo. Bacon, 1888. *Lib. Amer. Lit.*, Vol. XI, p. 412, Chas. L. Webster Co., N. Y., 1891) could possibly have written one single line of those plays. The *probabilities,* at least, are largely that they were not written by a man of whom Dr. Wainwright says himself, "He attended grammar school until fourteen years of age: from that age to eighteen we know nothing of his life"; and again, "I can find no evidence that he ever was [a student of medicine]." Dr. Wainwright quotes Lord Coleridge. But he forgets that Coleridge exclaimed: "Are we to have miracles in sport? Does God choose idiots by whom to convey divine truths to men?" And Emerson, "I can not marry this fact [lack of education] to his verse." Theo. Bacon asserts [*loc. cit.*], "Nor is there to be found in all the world of this profuse and voluminous author, of this bosom friend of poets, painters and actors, so much as the scratch of a pen on paper, except the three signatures upon his will, wherein by an interlineation which shows that he had at first overlooked the wife of his boyhood, he leaves her his "second best bed"; * * * there is nothing but the mean and sordid will to show that he ever put pen to paper.

But this matters not. It detracts not a whit from the value of Dr. Wainwright's meritorious and painstaking labor in winnowing out, isolating and presenting in attractive form the many gems relating to medicine. Every physician who really loves his profession, and who sees something in it besides money-getting possibilities; who loves knowledge, who loves literature, who would embelish his conversation, addresses and writings with gems of rhetoric and classical lore, should have the book. It is a treasure. I prize it very highly. Published by the author, 1907, Jno. W. Wainwright, M. D., New York City. Price not stated.

